# Quality of life and use of medication in chronic allergic and non-allergic rhinitis patients

**DOI:** 10.1186/2045-7022-3-S2-O4

**Published:** 2013-07-16

**Authors:** Christine Segboer, Carlijn Holland, Suzanne Reinartz, Ingrid Terreehorst, Artur Gevorgyan, Peter Hellings, Cornelis van Drunen, Wytske Fokkens

**Affiliations:** 1 Academic Medical Center Amsterdam, Otorhinolaryngology, Amsterdam, the Netherlands; 2 University Hospital Leuven, Otorhinolaryngology, Leuven, Belgium

## Introduction

In contrast to the significant number of studies indicating the impairment of QoL in AR, the degree of impairment in Quality of Life (QoL) in NAR remains underexposed. Again in contrast to AR, almost no evidence-based therapies for NAR patients exist. We assessed QoL in NAR compared to healthy controls and AR patients as positive controls and investigated whether the use of treatment in patients with NAR and AR had effect on QoL.

## Methods

An observational cohort study with 585 AR and 408 NAR patients was performed. Patients filled in the mini-RQLQ, assessing QoL related to symptoms of rhino-conjunctivitis. For the purpose of validation of the mini-RQLQ in NAR patients, 35 healthy controls working at the Otorhinolaryngology department were recruited. Both AR and NAR were defined as two or more of the following symptoms for > 1 hour on most days: watery, anterior rhinorrhea, (paroxysmal) sneezing, nasal obstruction, nasal pruritus and/ or conjunctivitis. For AR, these clinical findings had to be combined with one or more positive results on skin prick testing, relevant to the symptoms of rhinitis and/or conjunctivitis. For NAR, these clinical findings had to be combined with negative skin prick test results. A factor analysis assessing influence of age, gender, ARIA and use of medication on QoL was performed.

## Results

Validation of the mini- RQLQ for NAR showed that this questionnaire is able to discriminate between NAR and healthy controls. Analysis of a total of 111 NAR patients compared to 167 AR patients, showed a significant higher impairment of QoL in NAR compared to AR patients, both on overall symptom score as on different sub domains. The mean overall symptom score (2.45) of NAR patients was significantly higher (p = 0.002) compared to the overall symptom score of AR patients (1.94). A factor analysis showed no influence of any factors assessed on QoL, including use of medication.

## Conclusion

NAR patients had a significant higher impairment of QoL compared to AR patients. Use of medication did not influence QoL in AR and NAR.

